# Correction to: Green drugs in the fight against *Anisakis simplex*—larvicidal activity and acetylcholinesterase inhibition of *Origanum compactum* essential oil

**DOI:** 10.1007/s00436-018-5825-7

**Published:** 2018-03-12

**Authors:** Víctor López, María Cascella, Giovanni Benelli, Filippo Maggi, Carlota Gómez-Rincón

**Affiliations:** 1 Department of Pharmacy, Faculty of Health Sciences, Universidad San Jorge, Villanueva de Gállego, 50830 Zaragoza, Spain; 2 School of Pharmacy, University of Camerino, Camerino, Italy; 3 Department of Agriculture, Food and Environment, University of Pisa, Via del Borghetto 80, 56124 Pisa, Italy; 4 The BioRobotics Institute, Scuola Superiore Sant’Anna, Viale, Rinaldo Piaggio 34, Pontedera, 56025 Pisa, Italy

**Correction to: Parasitology Research**

**https://doi.org/10.1007/s00436-018-5764-3**

The article Green drugs in the fight against *Anisakis simplex*—larvicidal activity and acetylcholinesterase inhibition of *Origanum compactum* essential oil, written by Víctor López, María Cascella, Giovanni Benelli, Filippo Maggi, Carlota Gómez-Rincón, was originally published electronically on the publisher’s internet portal (currently SpringerLink) on 24 January 2018 without open access.

With the author(s)’ decision to opt for Open Choice the copyright of the article changed on 05 March 2018 to © The Author(s) 2018 and the article is forthwith distributed under the terms of the Creative Commons Attribution 4.0 International License (http://creativecommons.org/licenses/by/4.0/), which permits use, duplication, adaptation, distribution and reproduction in any medium or format, as long as you give appropriate credit to the original author(s) and the source, provide a link to the Creative Commons license and indicate if changes were made.

